# Elaboración y evaluación de un antisuero para la detección inmunohistoquímica del virus del Zika en tejidos fijados en aldehídos

**DOI:** 10.7705/biomedica.7857

**Published:** 2026-08-20

**Authors:** Ladys Sarmiento-Lacera, Gerardo Santamaría, Claudia Yadira Gómez, Alicia Rosales, Taylor Díaz, Diego Álvarez, Sheryll Corchuelo, Jorge Rivera, Alejandra Muñoz, Luz Mila Pinzón, Aura Caterine Rengifo, Verónica del Pilar Rincón, Orlando Torres-Fernández

**Affiliations:** 1 Grupo de Morfología Celular, Instituto Nacional de Salud, Bogotá, D. C., Colombia Instituto Nacional de Salud Bogotá D. C Colombia; 2 Grupo Genómica de Microorganismos Emergentes, Instituto Nacional de Salud, Bogotá, D. C., Colombia Instituto Nacional de Salud Bogotá D. C Colombia; 3 Grupo Animales de Laboratorio, Instituto Nacional de Salud, Bogotá, D. C., Colombia Instituto Nacional de Salud Bogotá D. C Colombia; 4 Grupo Aseguramiento de la Calidad, Instituto Nacional de Salud, Bogotá, D. C., Colombia Instituto Nacional de Salud Bogotá D. C Colombia; 5 Empresa Colombiana de Productos Veterinarios- VECOL, Bogotá, D. C., Colombia Empresa Colombiana de Productos Veterinarios- VECOL Bogotá D. C Colombia

**Keywords:** virus del Zika, sueros inmunes, inmunohistoquímica, histología, aldehídos, Zika virus, immune sera, immunohistochemistry, histology, aldehydes

## Abstract

**Introducción.:**

El sistema nervioso es uno de los blancos del virus del Zika. Es fundamental disponer de reactivos biológicos para apoyo diagnóstico e investigaciones sobre patogénesis que requieren tejidos fijados en aldehídos.

**Objetivo.:**

Determinar la utilidad de dos antisueros elaborados para la detección de antígenos del virus del Zika en células y tejidos fijados en aldehídos.

**Materiales y métodos.:**

Los antisueros se produjeron en conejos Nueva Zelanda a partir de un aislamiento viral del brote de Zika del 2016 en Colombia. En uno de los animales se empleó adyuvante de Freund para los refuerzos y, en el otro, el adyuvante Montanide. Se realizaron titulaciones por inmunorreacciones en células y tejidos positivos para el virus del Zika. Se emplearon los métodos de estreptavidina-fosfatasa alcalina y avidina-biotina-peroxidasa. Los antisueros se evaluaron en muestras infectadas experimentalmente para el virus del Zika y para otros virus relacionados y no relacionados; también, se evaluaron con muestras de otras técnicas utilizadas en el laboratorio, como cortes obtenidos con vibrátomo, muestras para microscopía óptica de alta resolución y microscopía electrónica de transmisión.

**Resultados.:**

Hubo inmunorreactividad a los antígenos del virus del Zika con ambos antisueros, principalmente en las neuronas de la corteza cerebral. Se demostró inmunotinción en las muestras de cultivos celulares y en los tejidos de animales infectados experimentalmente. Hubo inmunorreactividad cruzada con los virus del dengue y de la fiebre amarilla, pero no con el virus de la rabia.

**Conclusiones.:**

Se demostró la utilidad de los dos antisueros para la detección del virus del Zika en muestras de origen humano y de animales de experimentación, obtenidas mediante diferentes técnicas histológicas utilizadas para la investigación de la patogénesis de la enfermedad por el virus del Zika.

## Abstract (en)

**Introduction.:**

The nervous system is one of the main targets of the Zika virus. It is essential to have biological reagents to use as diagnostic support and in pathogenesis research that requires aldehyde-fixed tissues.

**Objective.:**

To determine the utility of two types of antiserums prepared for the detection of Zika virus antigens in cells and tissues fixed in aldehydes.

**Materials and methods.:**

The antiserums were produced in two New Zealand rabbits from a viral isolate from the 2016 Zika outbreak in Colombia. Freund's adjuvant was used for boosting in one of the animals, while Montanide adjuvant was used in the other. Immunoreaction titrations were performed on Zika-positive cells and tissues. The streptavidin-alkaline phosphatase and avidin-biotin-peroxidase methods were used. They were evaluated on experimentally infected samples for Zika and other related and unrelated viruses. Performance was evaluated with samples from other techniques used in the laboratory including vibratome sections, high-resolution optical microscopy samples, and transmission electron microscopy.

**Results.:**

There was immunoreactivity to Zika virus antigens with both antiserums, mainly in neurons of the cerebral cortex. Immunostaining was also demonstrated in cell culture samples and tissues from experimentally infected animals. There was cross-immunoreactivity with dengue and yellow fever viruses but not with rabies virus.

**Conclusions.:**

The utility of the two antiserums for the detection of Zika virus in samples of human origin and from experimental animals obtained by different histological techniques used for the investigation of Zika virus disease pathogenesis was demonstrated.

El virus del Zika (ZIKV) es transmitido principalmente por la picadura de mosquitos como *Aedes aegypti,* por transmisión vertical de la madre al feto o por vía sexual ^1^^,^^2^. La introducción del ZIKV en América, asociado a síndromes neurológicos en la población o ligado al síndrome congénito por el virus del Zika se convirtió en un gran reto para el continente ^3^.

En Colombia, el virus dejó consecuencias importantes, incluidas más de 18 000 mujeres gestantes expuestas, 365 niños con defectos asociados con el síndrome congénito por el virus del Zika y 16 597 nacidos vivos sin este síndrome que, en el seguimiento de años posteriores, reportaron alteraciones en el desarrollo de la audición y el lenguaje ^4^. En varios estudios recientes, se han descrito otras secuelas neurológicas en los niños de madres infectadas, aun sin haber presentado signos aparentes de la enfermedad por el virus del Zika al nacer ^3^.

En nuestro laboratorio, realizamos investigaciones básicas para estudiar el efecto que produce la infección por el ZIKV sobre el neurodesarrollo, evaluando la citomorfología y la neurobioquímica a partir de modelos *in vivo* e *in vitro* ^5^^-^^8^. Algunos de estos estudios requieren que se preserve la morfología y la inmunorreactividad de los marcadores de interés. En estos casos, se emplea fijación en aldehídos para establecer la correlación entre la presencia de material de origen viral en diferentes áreas del sistema nervioso y su efecto sobre los marcadores neuronales por medio de métodos inmunohistoquímicos. Esto permite el estudio de la patogénesis de esta infección mediante la evaluación de la citoarquitectura neuronal y la expresión tisular de diferentes proteínas ^7^^,^^8^.

Este tipo de estudios requieren la disponibilidad de gran cantidad de antisuero reactivo contra el ZIKV, que es escaso y costoso pues la mayor parte de la oferta comercial de anticuerpos está dirigida al diagnóstico del ZIKV en fluidos ^9^, no en tejido fijado en aldehídos, el cual forma puentes con las proteínas en el tejido, enmascarando al antígeno. Por esta razón, fue necesaria la elaboración de un antisuero y la posterior evaluación de su efectividad en:

- muestras de humanos o biomodelos infectados con el virus incluidas en parafina,
- muestras preservadas en aldehídos y
- muestras procesadas para microscopía óptica de alta resolución o microscopía electrónica de transmisión.

Hace algunos años llevamos a cabo un trabajo similar para obtener un antisuero antirrábico ^10^, que aún continuamos utilizando en nuestro laboratorio ^11^^,^^12^. Sin embargo, para la elaboración de este nuevo antisuero se incluyeron algunos ajustes al protocolo, en particular, porque se utilizó un virus vivo que fue necesario inactivar con luz ultravioleta, y se hizo un estudio comparativo del uso de dos adyuvantes.

## Materiales y métodos

### Animales utilizados

Se emplearon dos conejos machos de la raza Nueva Zelanda, de 12 semanas de nacidos y 2500 g de peso, mantenidos en el bioterio de barrera del Instituto Nacional de Salud. Para el adecuado manejo de los animales, se tuvieron en cuenta las normas vigentes en el país (Ley 84 de 1989 y Resolución 8430 de 1993 del Ministerio de Salud), así como las normas internacionales para el uso de animales de laboratorio en investigación ^13^.

El protocolo fue aprobado por el Comité de Ética y Metodologías de Investigación del Instituto, según el Acta No. 11 del 18 de mayo del 2021.

### Virus, adyuvantes y esquema de inoculación

Se empleó una cepa del ZIKV aislada de una paciente durante la epidemia en Colombia, identificada como zika_virus_459148_Meta_ Colombia_2016 (MH544701.2) ^6^. El virus se cultivó en células Vero; se ajustó a una concentración de 10^6^ unidades formadoras de placas (*plaque-forming unit,* PFU) y se inactivó con luz ultravioleta durante 14 minutos ^14^.

La suspensión viral se mezcló en partes iguales con adyuvante de Freund completo (Sigma ref. F5881) hasta obtener una emulsión que se usó para una primera inoculación de los conejos. La concentración final del virus en el inóculo fue de 5 x 10^5^ PFU, la cual fue empleada en cada una de las sucesivas inoculaciones.

Para la segunda fase de inoculaciones (realizada quince días después), se prepararon dos mezclas del siguiente modo: una parte de dilución del virus con una parte de adyuvante de Freund incompleto (Sigma ref. F5506), que se inoculó en uno de los conejos (conejo 1), y una parte de dilución viral con una parte de adyuvante Montanide™ (SEPPIC ref. ISA 55), que se inoculó en el otro conejo (conejo 2). Esto con el fin de poder comparar el desempeño en una reacción inmunohistoquímica de los antisueros obtenidos con cada adyuvante.

Cada mezcla se inoculó por vía subcutánea en el lomo de los conejos, sin sedación, aplicando a cada uno de ellos un volumen total de 0,5 ml, repartidos en 5 inyecciones de 0,1 ml, en áreas distantes una de otra.

Posteriormente, se aplicaron nuevas dosis del antígeno (dilución viral con adyuvante incompleto de Freund para el conejo 1 y dilución viral con adyuvante Montanide^TM^ para el conejo 2) a los 30 y 45 días después de la primera inoculación, para un total de cuatro inoculaciones.

Quince días después de la última inoculación de antígeno, se extrajo la sangre total por punción intracardiaca en condiciones de anestesia profunda, para lo cual se utilizó una combinación de ketamina (80 mg/kg) y xilaxina (8 mg/kg) inoculada por vía intraperitoneal 15. Luego, se obtuvieron los sueros mediante centrifugación a 3000 rpm durante 10 minutos a temperatura ambiente (20 °C).

### Titulación de antisueros

Los sueros extraídos de cada uno de los conejos se sometieron a temperaturas de 56 °C durante una hora para lograr la inactivación del complemento, y se distribuyeron en alícuotas que fueron mantenidas a -20 °C hasta su utilización. El mismo procedimiento se siguió con una muestra de suero control negativo, obtenido de los conejos antes de iniciar las inoculaciones.

Se realizó un estudio cualitativo en donde se registró la presencia o ausencia de inmunomarcación en tejidos y en células positivas para el ZIKV, empleando el método inmunohistoquímico de estreptavidina-fosfatasa alcalina ^5^^,^^16^^,^^17^. Para esto, las láminas fueron desparafinizadas y sometidas a recuperación antigénica con desenmascarante tris/EDTA pH 9 de Dako, durante 30 minutos al calor y, posteriormente, tres lavados sin agitación en TBS, cada uno durante 3 minutos.

Se bloqueó la fosfatasa alcalina endógena en ácido acético glacial al 20 % a 4 °C por 45 minutos. Luego de los tres lavados en solución tampón tris salino (TBS), se realizó el bloqueo de reacciones inespecíficas, incubando con un suero normal por 30 minutos que luego fue retirado sin lavar, para aplicar las diferentes diluciones seriadas de los dos tipos de antisueros (obtenidos con cada uno de los dos protocolos diferenciados por los adyuvantes utilizados), de acuerdo con la siguiente secuencia: 1:50, 1:100, 1:200, 1:400, 1:800, 1:1600, 1:3200 y 1:6400. Después de la incubación con los antisueros por una hora, se hicieron los tres lavados con la TBS para proceder a incubar por 1 hora con un anticuerpo secundario anti-conejo marcado con biotina a una dilución de 1:200.

Después de lavar tres veces con TBS, se incubó por una hora con estreptavidina marcada con fosfatasa alcalina a una dilución de 1:50. Después de tres lavados, la reacción fue revelada con el kit cromógeno Warp Red™ (Biocare wr8065) durante 3 minutos. El título se determinó como la dilución más alta que mostró marcación con el mínimo de fondo posible.

Para la titulación se emplearon las siguientes muestras, resumidas en el cuadro 1:

- una muestra de cerebro de ratón inoculado con ZIKV, correspondiente a material de archivo incluido en parafina, con resultado positivo previo por inmunohistoquímica efectuada con un anticuerpo específico donado por los *Centers for Disease Control and Prevention* (CDC) ^5^;
- una muestra de tejido humano positivo para ZIKV, correspondiente a material de archivo de patología, con resultado positivo previo por inmunohistoquímica efectuada con el anticuerpo donado por los CDC, así como diagnóstico con PCR, y
- una muestra de células Vero, infectadas con ZIKV, con resultado positivo previo por inmunocitoquímica efectuada con el anticuerpo donado por los CDC.

**Cuadro 1:** Resumen de muestras empleadas

Prueba	Tipo de muestra o prueba
Titulación	Material fijado con paraformaldehído e incluido en parafina:
	• tejido de ratón inoculado con virus del Zika,
	• tejido humano positivo para virus del Zika o
	• células Vero infectadas con virus del Zika.
Inmunorreactividad en muestras control negativo y positivo	Material fijado con paraformaldehído e incluido en parafina:
	• muestras de cerebro de ratón y células sin infectar o
	• muestras de cerebro de ratón y células infectadas.
Inmunorreactividad en muestras positivas para otros virus	Material fijado con paraformaldehído e incluido en parafina:
	• muestras de cerebro de ratón positivo para el virus del dengue,
	• tejido positivo para el virus de fiebre amarilla y
	• tejido positivo para el virus de la rabia.
Inmunorreactividad en muestras para probar la dilución elegida	Material fijado con paraformaldehído e incluido en parafina:
	• tejido de ratón (24 muestras) inoculado con virus del Zika o
	• tejido de ratón (10 muestras) no inoculado.
Inmunorreactividad en cortes de tejido obtenido con vibrátomo	• Tejido de ratón infectado, fijado con paraformaldehído y cortado con vibrátomo
Inmunorreactividad en células incluidas en resina acrílica LR White™	• Células infectadas, fijadas con glutaraldehído y procesadas para microscopía óptica de alta resolución y para microscopia electrónica de transmisión

### Pruebas del antisuero en otras muestras

El método de estreptavidina-fosfatasa alcalina se aplicó para los siguientes tejidos control (positivos y negativos), con la dilución óptima seleccionada mediante la prueba de titulación (cuadro 1):

- muestras de cerebro de ratón y células Vero sin infectar con virus;
- muestras de cerebro de ratón y células Vero infectadas con ZIKV;
- material de archivo experimental incluido en parafina y positivo para dengue, y
- material de archivo incluido en parafina y positivo para fiebre amarilla y rabia.

Finalmente, la dilución elegida también se probó en un total de 24 tejidos (nervioso o de diferente origen) correspondientes a muestras de archivo incluidas en parafina de un estudio previo sobre el el virus del Zika con material de ratón que había sido inoculado con el virus y 10 muestras control de material de ratón que no fue inoculado.

Los antisueros se probaron también en cortes de cerebro de ratón obtenidos con vibrátomo, de 50 μm de espesor, procesados por el método de avidina-biotina-peroxidasa (ABC), estandarizado en nuestro laboratorio para emplear en cortes de vibrátomo y correspondiente a material proveniente de otro proyecto de investigación sobre la enfermedad del virus del Zika del grupo ^7^. De este mismo material se utilizaron muestras incluidas en la resina hidrofílica LR White (Polysciences™) y procesadas para microscopía óptica de alta resolución e inmunomicroscopía electrónica de transmisión, según protocolos previamente estandarizados ^18^^,^^19^.

## Resultados

En las muestras del material incluido en parafina, el título óptimo del antisuero producido empleando solo el adyuvante de Freund fue de 1:200. El título ideal producido utilizando el adyuvante Montanide fue de 1:100. Este último produjo una positividad con menor ruido de fondo (figuras 1 y 2).

**Figura 1:**
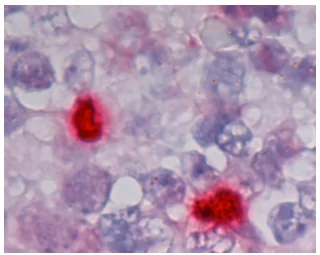
Neuronas en un corte de cerebro humano incluido en parafina. La marcación en rojo indica inmunorreactividad a antígenos del virus del Zika. Técnica de estreptavidina con fosfatasa-alcalina, 40X.

**Figuras 2A y B:**
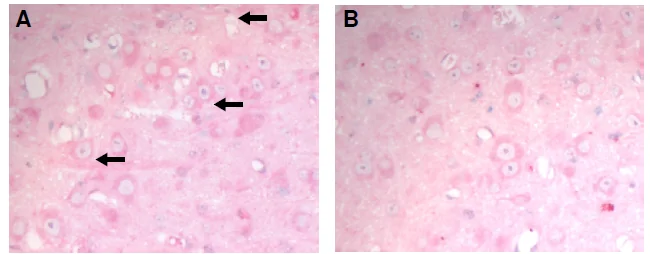
Neuronas identificadas por su núcleo voluminoso, en un corte de cerebro de ratón incluido en parafina. El fondo se observa con un tono más rosado para el adyuvante de Freund (A), comparado con el tono más suave para el ayudante de Montanide (B). Técnica de estreptavidina con fosfatasa-alcalina, 50X.

Las muestras de control positivo evaluadas con los dos antisueros correspondientes a cerebros de ratones y células Vero infectadas presentaron inmunorreactividad, mientras que en las muestras de control negativo correspondientes a cerebros de ratón sin infectar y células Vero no infectadas no se observó marcación.

En el presente estudio, se evidenció inmunomarcación en 23 de las 24 muestras analizadas que correspondían a reacciones inmunohistoquímicas con el antisuero obtenido con el adjuvante de Freund y, por otra parte, las mismas muestras procesadas con el antisuero obtenido con el adjuvante Montanide. En el tejido cerebral de origen humano, se observó el antígeno viral en el citoplasma de neuronas, principalmente las de la corteza cerebral (figura 1), en células del sistema inmunitario alrededor de los vasos sanguíneos, probablemente macrófagos, y en el endotelio. En las células Vero infectadas con el virus, se observó una fuerte inmunorreactividad con un título de 1:3200.

Se detectó reacción cruzada en muestras de hígado humano infectadas con el virus del dengue (DENV) y el de la fiebre amarilla (YFV), mientras que no se observó marcación en muestras de tejido nervioso infectadas con el virus de la rabia.

Fue notoria la diferencia entre el ruido de fondo que presentaban las láminas, dependiendo de si estas habían sido procesadas con el antisuero obtenido con el adjuvante de Freund o con el Montanide. El ruido de fondo fue menor con este último, como lo indica la densitometría (cuadro 2).

**Cuadro 2:** Densitometría óptica de la inmunorreactividad a antígenos del virus del Zika en cortes de cerebro de ratón, empleando el antisuero obtenido con el adyuvante de Freund y comparándolo con el obtenido con el adyuvante Montanide. Se empleó el programa ImageJ. Al medir la intensidad media de la luz transmitida (en una escala de 0 a 255), esta es mayor en la inmunohistoquímica procesada con el antisuero obtenido con el adyuvante Montanide, lo que indica una menor densidad del ruido de fondo, comparado con la que se observa en la inmunohistoquímica procesada con el antisuero obtenido con el adyuvante de Freund.

Número de mediciones en una lámina	Antisuero obtenido con el adyuvante de Freund	Antisuero obtenido con el adyuvante Montanide
1	171,652	206,118
2	195,096	227,185
3	179,625	211,042
4	188,662	227,085
5	170,786	243,488
6	176,723	218,050
7	195,915	231,823
8	175,685	225,199
9	176,231	244,939
10	186,762	204,962
Promedios	181,714	223,989

Los antisueros aplicados a cortes de cerebro de ratón de 50 μm de espesor, obtenidos en vibrátomo, mostraron reacción fuertemente positiva a una dilución de 1:2000 (figura 3). La reacción de los antisueros en muestras procesadas para microscopía óptica de alta resolución fue positiva a las mismas diluciones de 1:200 y 1:100 de las muestras incluidas en parafina (figura 4). Finalmente, en las muestras de células infectadas y procesadas para microscopía electrónica de transmisión se evidenciaron depósitos de partículas de oro coloidal en el citoplasma como una señal de inmunomarcación a una dilución de 1:200.

**Figura 3:**
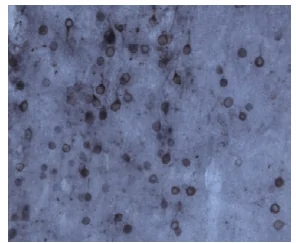
Imagen en blanco y negro de un fragmento de corte coronal de corteza cerebral de ratón, obtenido con vibrátomo. La tinción oscura (la original es café) del pericarion (citoplasma) y el núcleo vesicular grande (no reactivo) indica morfología de neuronas inmunorreactivas al virus del Zika. En algunas de ellas es evidente el segmento inicial de la dendrita apical. Técnica de avidina-biotina-peroxidasa y revelado con diaminobencidina-níquel, 20X.

**Figura 4:**
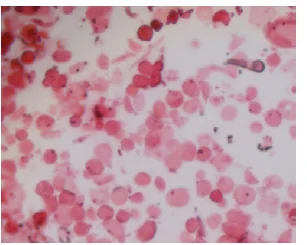
Células infectadas incluidas en resina hidrofílica y procesadas para microscopía óptica de alta resolución. Distintos grados de inmunorreactividad a antígenos del virus del Zika. Técnica de estreptavidina con fosfatasa-alcalina, 50X.

## Discusión

Se obtuvo un antisuero que detecta antígenos del ZIKV en material fijado en formaldehído o glutaraldehído y procesado mediante diferentes técnicas histológicas. Esta diversidad de análisis con diferentes métodos de fijación es importante dado que un determinante antigénico debe estar fácilmente disponible para que se produzca la unión eficiente entre antígeno y anticuerpo, pero el proceso de fijación puede desnaturalizarlo o enmascararlo y afectar su detección ^20^.

En el caso del glutaraldehído, fijador necesario para obtener una imagen de buena calidad en el microscopio electrónico, se sabe que afecta de manera negativa la detección inmunohistoquímica ^21^^,^^22^. El antisuero antirrábico que habíamos obtenido anteriormente también ha sido de utilidad en las muestras fijadas con aldehídos, incluido el glutaraldehído en concentraciones menores del 0,5 % ^10^^,^^11^.

La reacción cruzada en los tejidos infectados con los virus DENV y YFV, se explica por la estrecha relación que existe entre estos flavivirus ^22^^,^^23^. Se ha determinado, mediante comparaciones de similitud entre las secuencias de aminoácidos del ZIKV y otros flavivirus, una relación del 40 % de similitud para la proteína E, que puede variar según sea el flavivirus. Además, se ha descrito que algunos flavivirus se pueden organizar en serocomplejos debido a su característica de neutralización cruzada.

Las comparaciones de aminoácidos de la proteína E demuestran que el ZIKV ocupa una posición intermedia entre el YFV y el DENV ^24^. Es probable que esa relación entre la proteína y los virus pueda estar asociada con los resultados observados de inmunorreactividad cruzada para YFV y DENV con el suero anti-ZIKV, reconociendo aquellas regiones de aminoácidos compartidos por estos tres flavivirus. Esto sería útil si se quiere hacer una detección inicial para saber si una muestra contiene un flavivirus, para lo cual estos antisueros son sensibles y, a continuación, identificarlo con un anticuerpo específico para el virus en particular.

Por el contrario, con el tejido nervioso infectado con el virus de la rabia, correspondiente a un grupo taxonómico diferente, no se presentó reacción cruzada, lo que indica especificidad frente a otra enfermedad viral del sistema nervioso central.

Las variaciones observadas en los títulos dependen de factores como el tipo de procesamiento de la muestra, de si esta se incluyó o no en parafina o de si se utilizó la inmunohistoquímica en células o tejidos.

Los dos tipos de antisuero (según el adyuvante utilizado) presentaron un buen comportamiento porque lograron detectar los antígenos virales independientemente del adyuvante empleado. Se observó una reacción con menor ruido de fondo en la detección inmunohistoquímica hecha con el antisuero obtenido a partir del adyuvante Montanide. Otra ventaja reportada con el uso de Montanide en comparación con el adyuvante de Freund incompleto, está relacionada con menores efectos secundarios para los animales ^25^.

Es importante conocer los componentes del adyuvante y sus proporciones para obtener un buen producto que esté libre de contaminantes que ocasionen problemas graves de toxicidad en el sitio de inoculación ^26^^,^^27^. Según la información del fabricante del Montanide, el adyuvante contiene un aceite mineral del que no se indica su composición y un emulsionante extremadamente refinado obtenido de manitol con ácido oleico purificado de origen vegetal.

El adyuvante de Freund incompleto contiene aceite de parafina (aceite mineral) y, al parecer, un emulsionante que corresponde con la descripción encontrada para el de Montanide. La posible diferencia en el aceite mineral empleado o en el grado de refinamiento del emulsionante, podría ser uno de los factores que influyó en la obtención de reacciones inmunohistoquímicas con menor ruido de fondo o más limpias, como las producidas cuando se empleó el Montanide como adyuvante para la obtención de los antisueros. Se necesitarían más estudios para determinarlo.

No encontramos reportes que evidencien la obtención de una marcación inmunohistoquímica con tan escaso ruido de fondo al usar el Montanide como la obtenida por nosotros, comparada con la obtenida empleando el adyuvante de Freund incompleto, por lo que este sería el primer reporte.

La producción de estos antisueros en conejos y no en ratones de experimentación en los que serán utilizados, ofrece una ventaja ya que se evitarían las reacciones entre los anticuerpos circulantes propios del ratón, presentes en la muestra, el anticuerpo primario elaborado en ratón que se adiciona, y el anticuerpo secundario antiratón biotinilado que tendría que emplearse y que generaría ruido de fondo al unirse a los dos primeros

En conclusión, los antisueros anti-Zika obtenidos en conejos han sido y serán de utilidad para el trabajo experimental en ratones o muestras humanas de archivo, en las cuales las especificaciones son menos exigentes que para en el diagnóstico rutinario. Estos antisueros elaborados en conejos disminuyen la posibilidad de reacciones que generan ruido de fondo, cuando los antisueros se producen en la misma especie sobre la cual serán utilizados.
